# Community-wide benefits of targeted indoor residual spray for malaria control in the Western Kenya Highland

**DOI:** 10.1186/1475-2875-9-67

**Published:** 2010-03-03

**Authors:** Guofa Zhou, Andrew K Githeko, Noboru Minakawa, Guiyun Yan

**Affiliations:** 1 Program in Public Health, College of Health Sciences, University of California, Irvine, CA 92697-4050, USA; 2 Climate and Human Health Research Unit, Kenya Medical Research Institute, Kisumu, Kenya; 3 Department of Medical Entomology, Institute of Tropical Medicine and the Global COE Programme, Nagasaki University, Nagasaki, Japan

## Abstract

**Background:**

Interest in indoor residual spray (IRS) has been rekindled in recent years, as it is increasingly considered to be a key component of integrated malaria management. Regular spraying of each human dwelling becomes less and less practical as the control area increases. Where malaria transmission is concentrated around focal points, however, targeted IRS may pose a feasible alternative to mass spraying. Here, the impact of targeted IRS was assessed in the highlands of western Kenya.

**Methods:**

Indoor residual spray using lambda-cyhalothrin insecticide was carried out during the last week of April 2005 in 1,100 targeted houses, located in the valley bottom areas of Iguhu village, Kakamega district of western Kenya. Although the uphill areas are more densely populated, valleys are believed to be malaria transmission hotspots. The aim of the study was to measurably reduce the vector density and malaria transmission in uphill areas by focusing control on these hotspots. A cohort of 1,058 children from 1-5 yrs of age was randomly selected from a 4 km by 6 km study area for the baseline malaria prevalence survey after pre-clearing malaria infections during the third week of April 2005, and the prevalence of *Plasmodium *infections was tested bi-weekly. Seasonal changes in mosquito densities 12 months before the IRS and 12 months after the IRS was monitored quarterly based on 300 randomly selected houses. Monthly parasitological surveys were also carried out in the same area with 129-661 randomly selected school children of age 6-13 yrs.

**Results:**

The result of monthly parasitological surveys indicated that malaria prevalence in school children was reduced by 64.4% in the intervention valley area and by 46.3% in the intervention uphill area after 12 months of follow-ups in contrast to nonintervention areas (valley or uphill). The cohort study showed an average of 4.5% fewer new infections biweekly in the intervention valley compare to nonintervention valley and the relative reduction in incidence rate by week 14 was 65.4%. The relative reduction in incidence rate in intervention uphill by week 14 was 46.4%. *Anopheles gambiae *densities were reduced by 96.8% and 51.6% in the intervention valley and intervention uphill, respectively, and *Anopheles funestus *densities were reduced by 85.3% and 69.2% in the intervention valley and intervention uphill, respectively.

**Conclusion:**

Vector control had significant indirect impact on the densely populated uphill areas when IRS was targeted to the high-risk valleys. Additionally, the wide-reaching benefits of IRS in reducing vector prevalence and disease incidence was observed for at least six months following spraying, suggesting targeted IRS as an effective tool in malaria control.

## Background

Malaria epidemics have been frequently reported in the African highlands [1-3], and various malaria control measures have been implemented in different ecological settings [4]. Among those control measures, insecticide-treated bed nets (ITNs) and indoor residual-house spraying (IRS) are the two principle methods of preventing human-vector contact, thereby reducing malaria transmission [5-8]. The overall effectiveness of mass ITNs and IRS and their costs of implementation have been extensively studied [9-12]. The major burden for controlling malaria in the rural community is the cost and sustainability of the programmes [13-16].

An alternative way to reduce the cost of malaria programmes is targeted control. Since most previous ITNs and IRS trials have involved the indiscriminate dissemination of control measures, the effectiveness of targeted IRS is poorly understood, although barrier spraying for vector control has been reported as early as 1998 [17-19]. In terms of preventing transmission using IRS, mass spraying may be the only viable option to control vectors in the lowland endemic areas because every house in the clustered villages found on the flat plains of the lowland area is easily accessible to mosquitoes. In the highland regions of western Kenya, however, vector distribution can be markedly more heterogeneous. The highland region contains numerous valleys and basin-like depressions in a plateau where malaria transmission intensity ranges from low on hilltops to a level as high as that in the lowland in the valley bottoms [20]. A key determinant of this disparity is the location of aquatic vector breeding sites, which are generally confined to river banks and streams along the valley bottoms [21-23]. During the dry seasons, vector densities are low and human settlers in the valley areas are the main malaria reservoir and they maintain parasites year round. Indeed, the indoor density of *Anopheles gambiae *s.s. vectors, and malaria parasite prevalence decreases exponentially with the distance from the valley bottoms [21-25]. Shortly following the rainy seasons, more transient water bodies form in the uphill areas, thereby extending the range of the vector's breeding sites [22-24]. This unique setting provides an ideal site for effectively targeted IRS because insecticide spraying of houses in the valley areas before the onset of the wet season is expected to benefit residents of the uphill areas as well as in the valley.

## Methods

### Study design

**Figure 1:**
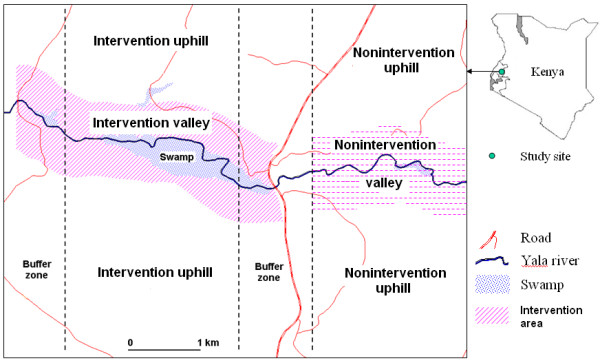
**Study area and experiment design**. Intervention and nonintervention valleys are defined as the area within 500 m of the Yala river in the intervention and nonintervention zones.

The targeted area is located in Iguhu village (0°17'N, 34°74'E, 1420-1580 m above sea level), Kakamega district, western Kenya. The detailed description of the study area has been included in an earlier study [23]. Briefly, the study area is 6.0 km by 4.5 km and is nearly equally bisected by the Yala river (Figure 1). The population is around 32,000 with 6,060 households (identified from 1 m Ikonos colour image). The targeted intervention area is within 500 m on both sides of the river with a length of 3.5 km from west to east (Figure 1). The study area was split into an intervention zone and a nonintervention zone that are separated by a 1 km buffer to ensure minimal adult mosquito dispersal between sites. For convenience, the actual sprayed valley area in the intervention zone is defined as "intervention valley" and the rest of the intervention zone is named as "intervention uphill", the corresponding areas in the nonintervention zone are defined as "nonintervention valley" and "nonintervention uphill" (Figure 1).

### Targeted IRS

All houses in the targeted intervention valley area were sprayed with lambda-cyhalothrin (ICON), which is among the insecticides recommended by the World Health Organization and National Malaria Control Board of Kenya. All houses in the intervention valley (total of 1,100 houses) were identified and numbered. The interior walls and roofs of the targeted houses were sprayed during the last week of April 2005. This period was prior to the long rainy season of May that triggers a high density of malaria vectors and the start of the peak malaria transmission period in this highland area. There was no other IRS or systematic use of ITNs existing in the study area. Field surveys in 2004 showed that household bed net ownership was about 13% (49/382) and bed net coverage was about 5% (98/1958) of the surveyed population (assuming that one bed net covers two individuals). No other bed net data was available for the study area from 2004 until the mass distribution of free bed nets and free artemisinin combination therapy (ACT) for children under five years of age in the study area commenced in July-September 2006.

### Sample size of the epidemiological survey

The sample size for the baseline survey was estimated in advance to have 90% power to detect a 10% reduction in malaria prevalence in children aged between one and five years, assuming a loss to follow-up of 15% during the 16 week survey, a two-sided type I error probability of 0.05, and a 1:1 ratio of intervention to control (Table 1).

**Table 1:** Study population and sample size for entomological and parasitological surveys by study areas.

	Intervention		Nonintervention		Buffer areas^†^
		
	Valley	Uphill	Valley	Uphill	
Approximate population	2800	8800	2200	7400	10800
Mosquito survey: Mean number of houses sampled per survey	24	79	29	65	103
Monthly parasite survey: Mean number of children sampled per survey	45	75	48	134	84
Cohort study: Mean number of children sampled per survey	113	154	112	249	321

^† ^Buffer areas include all buffer areas.

### Cohort study

A cross-sectional survey was conducted during the third week of April, 2005, before the IRS, to obtain the baseline malaria prevalence data. Finger prick blood samples were taken from 1,058 randomly selected children aged 1-5 yrs for detection of the *Plasmodium falciparum *antigen (Pf HRP-2) using the Optimal (DiaMed AG, Switzerland) rapid whole blood immunochromatographic test. Children found to be positive for malaria infection were given free anti-malarial treatment according to Kenyan guidelines. A cohort malaria incidence study was started after 14 days of IRS and a blood sample test was performed using the same Optimal rapid test kit every other week until week 16.

A monthly parasitological survey was carried out in the same area from May, 2004 to March 2006 with an average of 386 (range from 129 to 661) school children aged 6 to 14 yrs randomly surveyed each month. The detailed blood sample collection method including the blood smear preparation, parasite density determination and quality controls can be found in Munyekenye *et al *[20]. The number of children surveyed each month is shown in table 1.

Children were recruited into the study only with the informed consent of their parents or guardians. Scientific and ethical clearance was given by the Kenya Medical Research Institute (KEMRI) and State University of New York at Buffalo ethical review boards.

Only children under 14 yrs were included in this study because this group has been shown to be the most vulnerable to malaria parasite infection [20].

### Entomological survey

The required mosquito adult sample size has been estimated by an earlier study [23]. Indoor resting mosquitoes were collected from 300 houses in the study area by pyrethrum spray collection. Houses were selected to ensure maximal spatial coverage. The number of *Anopheles *mosquito females was recorded in the intervention zone and control zone. The indoor resting density was determined as the average number of *Anopheles *females collected for each house. The timings of four mosquito surveys were selected to represent the vector population during the different seasons both before IRS (May, August and November 2004 and February 2005) and after IRS (May, August, and November 2005 and February 2006). Sample sizes of each strata are shown in table 1.

Anophelines were morphologically identified and classified as *Anopheles gambiae *and *Anopheles funestus *using morphological keys [25]. Since earlier studies had shown that all *An. gambiae sensu lato (s.l.) *and *An. funestus s.l*. specimens were identified as *An. gambiae sensu stricto (s.s.) *and *An. funestus s.s*. by using the rDNA-polymerase chain reaction (PCR) method [25,26], no further classification work was conducted.

### Statistical analysis

Changes in seasonal mean vector densities and monthly parasite prevalence before and after intervention were tested by the use of planned comparison of GLM, using location (valley and uphill) and survey period (before and after intervention) as independent variables and using outcomes from the corresponding nonintervention area as contrast for the dependent variable. The differences in biweekly new infections and parasite prevalence between intervention and nonintervention valleys (or uphill) were tested by using Mantel-Cox test [26]. STATISTICA version 8.0 (StatSoft, Tulsa, OK) was used for data analysis.

Relative risk of *Plasmodium *parasite infection (*RR*) during the cohort study is defined as the ratio of *Plasmodium *parasite prevalence in the intervention valley (or uphill) over the parasite prevalence in the nonintervention valley (or uphill). The protective effectiveness of IRS in reducing malaria infection is measured as (1-*RR*) and the asymptotic confidence intervals were calculated as *RR*(1 ± z_α/2 _), where z_α/2 _is the upper α/2 percentile of the standard normal distribution and *u *is the variance of the ratio [27].

To assess changes in mosquito densities and *Plasmodium *parasite prevalence associated with the implementation of IRS, the percentage reductions (*PR*) in parasite prevalence and mosquito densities in targeted intervention valley and uphill areas was calculated using the following formula:

where *C*_1 _and *C*_2 _(*T*_1 _and *T*_2_) describe the average densities of mosquitoes or parasite prevalence in the control (targeted) zone during baseline (subscript 1) and intervention (subscript 2) periods. This formula takes into account that changes in the mosquito populations and parasite prevalence are taking place at the same level and rate in both targeted and control zones, i.e., the reductions were adjusted for the background differences.

## Results

### Baseline parasitological descriptive statistics

During the last week of April 2005, 1,058 children were recruited for a baseline malaria prevalence test and 1,033 of them were included in the final data analysis. The remaining children have at least one missing record and were not used for the cohort analysis. Among those who were included in the cohort study, 113 were from the intervention valley (Table 1) with 83 (73.5%) P. falciparum positive slides and 112 from the corresponding non-intervention valley with 49 (43.8%) parasite positive slides (Yates χ^2 ^= 12.5, d.f. = 1, P < 0.001). The relative risk was 1.5 (95% CI [1.2, 2.0]) and the odds ratio was 2.7 (95% CI [1.6, 4.5]). Similarly, the parasite prevalence was 61.7% (95/154) in the intervention uphill and it was significantly higher than the prevalence of 31.4% (77/249) in the nonintervention uphill (Yates χ^2 ^= 34.1, df = 1, P < 0.0001). The relative risk was 2.0 (95%CI [1.6, 2.5]) and odds ratio was 3.5 (95%CI [2.3, 5.4]).

### Temporal dynamics of *Plasmodium *prevalence

**Figure 2:**
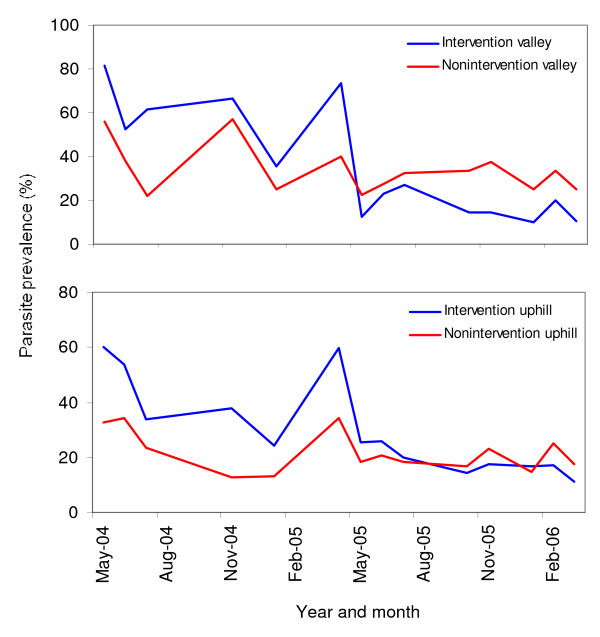
**Dynamics of parasite prevalence in different areas from April 2004 to March 2006**.

Among parasites detected, *P. falciparum *accounted for 97.2% of positive samples, *Plasmodium malariae *comprised 3.9% and *Plasmodium ovale *less than 2%. There were a few cases of mixed infections of two parasite species. The longitudinal follow up study shows that malaria parasite prevalence in children in both the intervention and nonintervention areas have decreased since May 2005 (Figure 2, Table 2), but the decrease was much higher in the targeted intervention area than in the nonintervention area. Parasite prevalence in the intervention valley dropped significantly from 63.6% before intervention to 16.4% after the intervention (GLM planned comparison, *F*_1,24 _= 309.29, *P *< 0.0001), the adjusted relative reduction in parasite prevalence was 65.4%. The relative risk of malaria parasite infection dropped in the intervention valley from an average of 1.7 (range from 1.2 to 2.8) before the intervention to 0.6 (range from 0.4 to 0.8) after the intervention. Concordantly, parasite prevalence in the intervention uphill area decreased from 44.9% before intervention to 18.5% after intervention (Figure 2, Table 2), representing an adjusted reduction of 46.4%, and relative risk of parasite infection decreased from an average of 1.9 (range from 1.4 to 3.0) to 1.0 (range from 0.6 to 1.4).

**Table 2:** Reductions in parasite prevalence and mosquito densities after IRS.

Location	Survey period	Intervention	Nonintervention	Adjusted reduction (%)^†^
*P. falciparum *prevalence (%)				
Valley	Before intervention	63.61 [53.63, 73.61]	39.71 [32.24, 47.18]	
Valley	After intervention	16.44 [7.81, 25.11]	29.62 [23.16, 36.09]	65.35
Uphill	Before intervention	44.86 [34.87, 54.85]	25.04 [17.57, 32.51]	
Uphill	After intervention	18.53 [9.88, 27.18]	19.28 [12.81, 25.74]	46.35
*An. gambiae *density (female/house/night)				
Valley	Before intervention	3.41 [1.04, 5.76]	2.06 [0, 5.14]	
Valley	After intervention	0.17 [0, 2.28]	3.18 [0.42, 5.93]	96.82
Uphill	Before intervention	0.94 [0, 3.31]	0.61 [0, 4.11]	
Uphill	After intervention	0.47 [0, 2.58]	0.63 [0, 3.05]	51.6
*An. funestus *density (female/house/night)				
Valley	Before intervention	0.78 [0.25, 1.31]	0.23 [0.06, 0.39]	
Valley	After intervention	0.06 [0, 0.54]	0.12 [0, 0.27]	85.26
Uphill	Before intervention	0.26 [0, 0.79]	0.08 [0, 0.25]	
Uphill	After intervention	0.05 [0, 0.53]	0.05 [0, 0.19]	69.23

† Reduction in parasite prevalence and vector densities in the intervention valley and uphill area after intervention were adjusted assuming no change in the corresponding nonintervention valley or uphill area.

### Incidence rate change in cohort study

**Figure 3:**
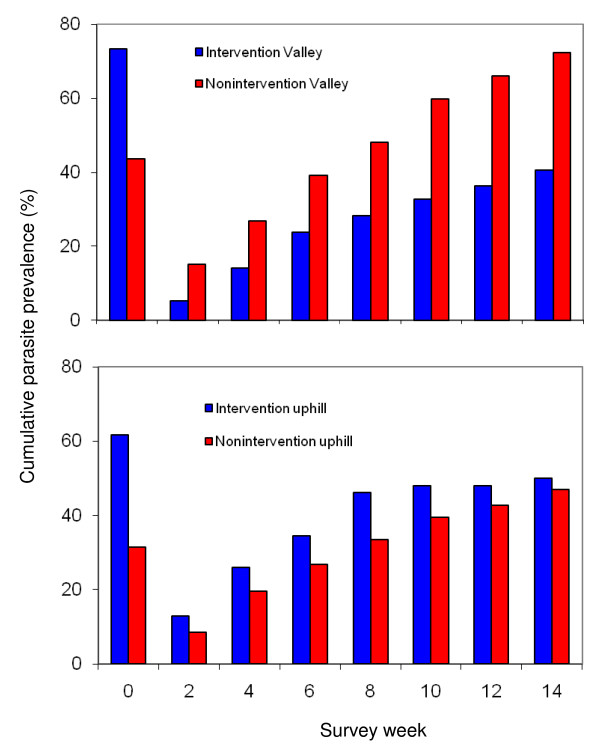
**Biweekly cumulative incidence rate and changes in relative risk in different areas**. Survey time week 0 (Wk 0 on x-axis) represents baseline survey. The relative risk was adjusted using baseline surveys as the unit between intervention and nonintervention valleys (or uphill areas).

Figure 3 shows that baseline parasite prevalence (week 0) and cumulative incidence rate in the subsequent biweekly surveys during the 14 weeks cohort study period. Parasite infections rebound significantly faster in the nonintervention valley than that in the intervention valley (Figure 3) (Mantel-Cox test, Z = 4.94, P < 0.001). On average, there were 4.5% fewer new infections (range from 1.8% to 9.9%) in the intervention valley than in the nonintervention valley. By week 14, the cumulative incidence rate reached 40.7% and 72.3% in the intervention valley and nonintervention valley, respectively, compared to the baseline prevalence of 73.4% and 43.8% in the corresponding areas. The adjusted reduction by week 14 was 65.4%.

While the cumulative incidence rate was significantly higher in the intervention uphill than nonintervention uphill (Figure 3) (Mantel-Cox test, Z = 7.30, P < 0.001), the average difference in biweekly new infection rates was insignificant (0.9%, range from -3.7% to 5.1%). By week 14, the cumulative incidence rate was 50.0% in the intervention uphill area, which was significantly lower than the baseline prevalence of 61.7%, whereas cumulative incidence rate was 46.9% in the nonintervention uphill area by week 14 compared to a baseline prevalence of 31.4%. A resulting adjusted reduction of 46.4% was calculated for the cumulative incidence rate in the intervention uphill.

### Temporal changes in vector abundance

**Figure 4:**
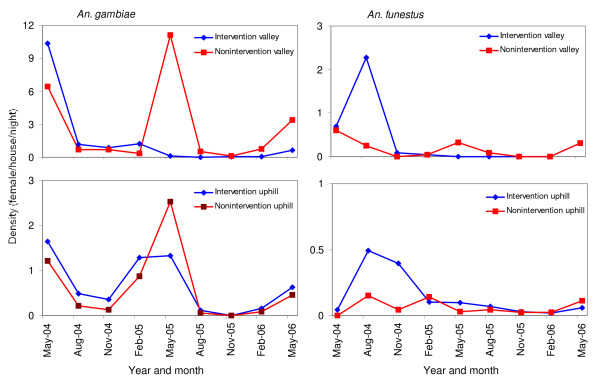
**Temporal changes in *An. gambiae *(left panel) and *An. funestus *(right panel) densities from May 2004 and February 2006**.

*Anopheles gambiae *density in the intervention valley was reduced more than ten-fold from an average of 3.4 female/house/night (f/h/n) before intervention to 0.2 f/h/n after the intervention (Figure 4) (GLM planned comparison, *F*_1,14 _= 7.63, *P *= 0.02), which was a 96.8% relative reduction (Table 2). The adjusted reduction in *An. gambiae *densities in the intervention uphill area was 51.6% (Table 2). Average density of *An. funestus *in the intervention valley was also significantly reduced from 0.78 f/h/n before intervention to 0.06 f/h/n after the intervention (Table 2) (GLM planned comparison, *F*_1,14 _= 9.16, *P *= 0.01), and the adjusted reduction was 85.3%. The relative reduction in *An. funestus *densities in the intervention uphill area was 69.2% (Table 2).

## Discussion

Because of their historical success, insecticide-treated bed nets (ITNs) and indoor residual spray (IRS) are among the principal malaria prevention and control measures in Africa. However, the high efficacy of indoor residual spraying in malaria control is usually offset by high costs in terms of logistics and the cost of insecticides. The present analysis represents a first for assessing the efficacy of targeted IRS with a cohort-design study [28]. The western Kenya highlands provide an excellent opportunity for targeted malaria control because more than 90% of the vectors are confined to a narrow band close to the valley bottoms [23-25]. Such an application is expected to have a mass effect in the unsprayed areas, i.e., the community-wide benefits [29,30], while reducing the costs involved in the spray programme. This targeted approach is applicable in many settings across Africa because much of the population of Africa lives in highland areas and even more of the population is aggregated around rivers, streams, lakes and swamps because land and fertile soil are limiting resources.

Vector control measures vary considerably in the scope of their applicability. The result of this trial yields an overall 50% reduction in malaria prevalence, which is comparable to the average effectiveness of mass IRS and ITNs [10]. An important advantage of this targeted IRS programme is the reduction in cost. A blanket application of IRS in the intervention zone would have covered at least 4,000 houses at a cost of US $14,444, not including indirect and logistical costs. However, since only 25% of the houses were sprayed in our study, the cost of insecticide was only US $3,611. It is estimated that the same proportional cost saving (and equivalent time saving) was achieved on transport and personnel budgets. Hence, a lasting and substantial reduction in malaria prevalence that is comparable to previous mass spray studies resulted from our targeted approach representing considerable financial savings.

One limitation of the study is the pyrethrum spray catches as a means to measure mosquito densities. Because of the implementation of IRS, mosquitoes might be expected to be less inclined to rest in treated houses after feeding, thus mosquito densities in IRS houses may be underestimated by PSC. In which case, the encouraging results that we obtained from comparison of mosquito densities before and after intervention in the uphill intervention sites would in actuality downplay the overall indirect effect of targeted spraying. Another major limitation is the number of sites tested since there was only one nonrandomized IRS experimental unit. More rigorous randomized trials are planned.

## Conclusions

Vector control remains the most generally effective measure to prevent malaria transmission. IRS has been proven as one of the most effective methods for controlling malaria transmission. Targeted IRS is most suitable for low endemic areas with focal malaria where it can greatly reduce programme cost with comparable effectiveness. Appropriate application or integration of IRS with other interventions, such as ITNs, elsewhere on the continent has to be based on sound scientific research which takes into account the ecological and epidemiological setting, organizational capacity, and social and financial considerations as these in turn impact on operational feasibility and sustainability. This trial study demonstrates the relative simplicity of substantial cost reduction by implementing more judiciously targeted control. In doing so, this study have demonstrated how this effective control tool might become more widely available either as a stand-alone measure or as part of an integrated malaria management programme.

## Competing interests

The authors declare that they have no competing interests.

## Authors' contributions

GZ assembled data, performed statistical analysis and drafted the paper. NM, AKG and GY designed the study, supervised data collection and drafted the paper. All authors read and approved the final manuscript.
